# Practical Guide to the Local Treatment of Sjogren’s Disease

**DOI:** 10.3390/jcm15031078

**Published:** 2026-01-29

**Authors:** Elizabeth J. Price, Shirleen Hallang, Guy Smith

**Affiliations:** 1 Great Western Hospital NHS Foundation Trust, Swindon SN3 6BB, UK; 2 Bristol Dental School, Bristol Dental Hospital, University Hospitals Bristol and Weston NHS Foundation Trust, University of Bristol, Bristol BS2 0PT, UK; shirleen.hallang@bristol.ac.uk

**Keywords:** Sjogren, sicca, mucosal, ocular, oral, vaginal, topical treatment, eye drops, dental care

## Abstract

Dryness (sicca) of mucosal surfaces is the hallmark of adult-onset Sjogren’s disease (SjD). In our experience, clinicians frequently lack confidence in managing the sicca symptoms effectively. Successful management requires an understanding of the condition and personalisation of care. We have reviewed the available evidence and, when necessary, used personal and expert experience to provide comprehensive advice on the management of ocular, oral, and systemic sicca, enabling the treating physician to support their patients and manage their symptoms effectively and prevent long-term damage. Our objective is to ensure that the non-specialist feels confident in managing the myriad manifestations of sicca in SjD.

## 1. Introduction

Oral and ocular dryness (sicca) are the usual presenting features of SjD and form part of the diagnostic criteria [1], but involvement of other mucosal surfaces is common.

Oral and nasal dryness reduces your ability to perceive taste and smell, and ocular dryness causes discomfort and blurring of vision. Both have a detrimental effect on the quality of life (QoL) [2]. Vaginal dryness [3] and atrophy [4] lead to a significant reduction in sexual health [5]. Systemic dryness contributes to a chronic dry cough and affects voice quality. Patients can suffer from skin rashes, dry skin, itching, and discomfort. Most of these manifestations respond poorly, if at all, to conventional disease-modifying drugs and need tailored treatments. Clinicians should aim to support individuals to manage their condition by preserving, replacing, and stimulating secretions and suppressing underlying systemic disease activity to prevent disease progression and damage. Our objective is to ensure that the non-specialist feels confident in managing the myriad manifestations of sicca in SjD.

This article focuses on the practical management of mucosal surface dryness. A fuller guide to the practical management of SjD is available at Guidelines | British Society for Rheumatology (https://www.rheumatology.org.uk/guidelines, accessed on 18 January 2026).

## 2. Management of Dry Eye

Patients with SjD suffer from a combination of aqueous tear deficiency and poor meibomian gland function, resulting in tear film instability together with ocular surface inflammation and epithelial degradation [6]. All these manifestations need to be adequately managed to prevent long-term damage. There is increasing interest in the use of tear film biomarkers as measures of inflammation. The presence of elevated matrix metalloproteinase-9 (MMP-9) in tears, which is available as a point-of-care test, enables early and accurate diagnosis of an inflamed dry eye, allowing earlier and targeted intervention [7]. Other biomarkers of interest include ICAM-1, IL-6, IL-1beta, IL-17a, and Tumour necrosis factor-alpha (TNF-alpha), with correlation between elevated levels and presence of active surface inflammation [8].

### 2.1. Lubricating Eye Drops to Replace Aqueous Tears

Tears and eye drops evaporate rapidly following instillation and studies have confirmed that those instilling eye drops regularly, rather than symptomatically, have significantly better patient-reported outcomes [9]. All patients with SjD-associated dry eye should be offered preservative-free lubricating eye drops and advised to instil them 2 to 3 hourly [10].

When choosing a suitable eye drop, it is critical to opt for a preservative-free formulation or one where the preservative degrades into harmless ingredients upon exposure to air or the corneal surface to avoid worsening of surface inflammation. The majority of lubricating eye drops are safe and effective, but comparisons between formulations are difficult due to the lack of consistency in trial design [11]. Lubricating eye drops improve ocular symptoms within four weeks of regular use, but signs may take longer to improve [12]. Combination eye drops may be more effective than single-ingredient drops [12].

It is important to personalise the eye-drop regimen to the patient. We recommend that when treating individuals with SjD dry eye, you should begin with a preservative-free sodium hyaluronate or hyaluronic acid (HA) containing drop four times daily alongside a more viscous gel or ointment last thing at night. In general, a meta-analysis of the efficacy of HA drops for dry eye [13] confirmed that they were more efficacious than simple saline or non-HA-based eye drops.

Carmellose-based drops may offer better retention in patients who find their drops offer only very short-lived relief. If there is a rapid tear break-up time (TBUT), then a poly vinyl alcohol (PVA)-based drop or a combination drop containing lipid may be beneficial in stabilising the tear film. If there is persistent discomfort or surface inflammation, then a combination drop with sodium hyaluronate and trehalose or Coenzyme Q10 may be of benefit.

Ointments are longer-acting than drops and are useful for overnight use. They can blur the vision and are usually paraffin-based and contain lanolin. Switch to a lanolin-free preparation if needed. Always choose a preservative-free product.

If the patient has filamentary keratitis with mucus stranding, then, in addition to the moisturising eye drops and ointments described above, consider using acetylcysteine eye drops, which act as a mucolytic [14].

It is important to bear in mind that formulations change frequently, and some drops and ointments may be out of stock from time to time because of manufacturing and supply issues. Substitute formulations may be needed, but always remember to ensure that a preservative-free option is chosen (See Table 1 for advice on which drop to use).

### 2.2. Serum Eye Drops

Blood-derived serum eye drops, either autologous or allogeneic, are well tolerated by patients, with a number of studies confirming an improvement in Ocular Surface Disease Index (OSDI), TBUT [15] and Rose Bengal staining score in those treated with serum eyedrops compared to lubricating eye drops [16]. A systematic review recently concluded that although there was evidence that serum eye drops improved symptoms, objective evidence of improvement in the corneal surface was lacking, and the effectiveness of serum eye drops was uncertain based on current data [17].

In the UK, serum eye drops are supplied by the Blood Transfusion Service and are only available when patients satisfy the relevant NHS commissioning policy statement. Many individuals struggle with donating blood because the autologous drops and allogeneic drops are expensive to produce and in limited supply.

### 2.3. Treating Corneal Surface Inflammation

A few studies have examined treating corneal surface inflammation associated with SjD with immunomodulating eye drops. Among these, only ciclosporin eye drops are routinely available in the UK, with evidence that they improve corneal staining (*p* < 0.05) and TBUT (*p* < 0.05) in aqueous dry eye of differing cause but are of less benefit in evaporative dry eye [18]. Consensus opinion allows for the use of ciclosporin eye drops in children from four years of age, based on extrapolated evidence from the literature [19], but there are no published studies in juvenile onset SjD (jSD).

A randomised clinical trial (RCT) of topical fluorometholone (FML) 0.1% eyedrops versus cyclosporin 0.05% eye drops in SjD found that both treatments improved objective signs and patient-reported symptoms of dry eye following eight weeks of treatment, with earlier improvement in the FML treated group [20].

Subsequently, Fondi et al. compared ciclosporin eye drops alone in combination therapy with hydrocortisone at initiation of the therapy. They found a significant benefit in the objective and patient-reported symptoms (*p* = 0.002) for both regimens at 2 weeks and 6 months of follow-up, with a trend (non-significant) to faster improvement in clinical symptoms and better outcomes at 6 months in the combination group [21].

Tacrolimus eye drops are available in the US and parts of mainland Europe, but not the UK. Tacrolimus 0.03% and cyclosporine 0.05% eye drops have been compared to each other in 60 individuals with SjD, acting as their own control [22]. Both preparations were equally effective at improving patient-reported symptoms and objective signs compared to the placebo-controlled eye.

Lifitegrast eyedrops are licenced in the Far East and the US but are not NICE approved nor available in the UK. Four multicentre RCTs (results summarised in [18]) showed a significant improvement in patient-reported symptoms and inferior corneal staining.

Rebamipide eye drops—a quinolone derivative—are not currently available in the UK or Europe but have been used in Japan since 2012. They have been shown to increase corneal and conjunctival mucin levels and stabilise the tear film, with a recent systematic review concluding that rebamipide ophthalmic suspension was safe and effective for the treatment of dry eyes and improved TBUT [23].

Diquafosol—a purinergic P2Y2 receptor agonist promoting fluid transfer and mucin secretions—is available in Japan, but not the UK or Europe, as a 3% ophthalmic solution. Studies show improvements in objective signs of dry eye disease [24] and it is recommended for use in adult SjD and jSjD within the Japanese guidelines [25].

In practice, we recommend the following approach in individuals with persistent surface inflammation despite adequate moisturising eye drops. We start with a dexamethasone 0.1% preservative free eye drop four times daily for seven days, three times daily for seven days, twice daily for seven days, once daily for seven days, and then stop. Alongside this, we introduce ciclosporin 1 mg/1 mL eye drops nocte in both eyes after the first week of dexamethasone and if tolerated and effective, continue long-term.

A few patients develop superimposed seasonal allergic symptoms, in which case a short course of Ketotifen eye drops may be helpful.

### 2.4. Treatments for Meibomian Gland Deficiency (MGD)

Meibomian glands within the eyelids produce a thin outerlayer of lipid which sits on the surface of the tear film, reducing tear evaporation and stabilising the tear film. Patients with meibomian gland deficiency experience increased tear evaporation and reduced TBUT. Stimulating the meibomian glands to improve lipid secretion improves comfort, slows tear evaporation, and improves TBUT.

All routinely available treatments for meibomian gland disease have been shown to be effective to a greater or lesser degree. These include self-applied eyelid warm eye compresses, thermal pulsation (Lipiflow**^®^**), Intense Pulsed Light therapy (IPL), meibomian gland (MG) intraductal probing, antibiotic treatment, combination eyedrops containing a lipid component, and pure oily eyedrops such asperfluorohexyloctane although clinical response varies [26].

Warm eye compresses (microwaveable or USB powered) are cheap, widely available, and simple to use, with studies confirming their benefit on symptoms and objective measures of tear film stability [26]. We recommend that our patients use one daily for at least 10 min.

A single session of Thermal pulsation (Lipiflow**^®^**) therapy has been shown to be sufficient to produce improvement in both the quality of meibomian gland secretions and patient-reported symptoms in those with MGD but no evidence of superiority to a basic warm compress in controlled trials [27]. Similarly, both IPL combined with meibomian gland expression (MGX) and IPL alone are safe and effective treatments for MGD [28]. There is some evidence that targeted use of IPL in patients with raised tear film IL-6 and IL-1beta may be more effective [8]. However, these treatments are only currently available in the UK within the private sector.

MG probing is performed under slit lamp guidance using a fine probe (approximately 80 μm wide and 2 mm long). Although many of the uncontrolled studies suggest benefit, the controlled studies do not show significant objective improvement [29]. It is only currently available in the UK via the private sector.

Antibiotic treatment (usually a low dose of doxycycline acting as both an anti-inflammatory and an anti-microbial) may result in short term improvements, but treatment may need to be continued long-term for sustained benefit [30].

### 2.5. Lipid-Containing Eye Drops

Lipid-containing eye drops work by replacing the absent or depleted lipid layer and have been shown to improve patients both symptomatically and objectively [26]. They are available as both a combination drop—usually combined with sodium hyaluronate—or as single ingredient drops, e.g., perfluorohexyloctane, or liposomal eye sprays. If using a spray or single ingredient drop the patient should be advised to instil them after their usual moisturising eye drop to replicate the natural tear layers.

### 2.6. Punctal Occlusion

Punctal plugs may improve symptoms in some patients despite the lack of evidence from clinical trials [31]. In practice, we consider punctal plugs where moisturising eye drops need to be instilled more than half hourly and where surface inflammation has been effectively treated. Patients should ideally have no residual corneal surface inflammation at the time of insertion. We would also recommend that temporary plugs are sited in the first instance, so they can be removed if symptoms worsen.

### 2.7. Androgen Replacement Therapy (ART)

Androgen Replacement Therapy (ART), either topically or via oral or transdermal application, may confer short-term benefit, but there is no evidence of long term benefit in dry eye disease [32] and side effects are common but usually mild.

### 2.8. Stimulatory Treatments for Ocular Sicca

Pilocarpine is a muscarinic acetylcholine agonist primarily targeting the M3 muscarinic receptors and stimulates exocrine secretory glands in a non-specific way. Pilocarpine can stimulate secretions in up to 70% of patients with sicca symptoms. Adverse effects are common but usually mild and predicted by the increase in parasympathetic activity. Most of the studies date from 20 years ago, with no new recent trials. Two large RCT’s (256 and 373 individuals) and a smaller unblinded study of 85 individuals with SjD showed significant improvement (*p* < 0.0001) in both symptoms and signs of dry eye [33,34,35]. Use of pilocarpine is often limited by side effects such as sweating and flushing. In practice, we manage this by starting with a low, once daily dose and titrating slowly upwards.

Cevimeline is a cholinergic agonist that targets both the muscarinic M1 and M3 receptors. It is available in the US (licenced only for adults) but not in Europe or the UK. There is weak evidence of a clinical benefit to cevimeline compared to placebo in dry eye [36,37].

There are a few studies suggesting intranasal neurostimulation may benefit some patients with dry eye disease, including those with SjD [38] and a commercial device is available in the USA.

### 2.9. Summary Recommendations for Ocular Sicca

- Start with preservative-free sodium hyaluronate-containing eye drops at a low percentage.
- Combine with a night-time eye ointment.
- Recommend use of a microwaveable or USB-heated warm compress for 10 min daily.
- If ongoing symptoms increase percentage of sodium hyaluronate drops or switch to a combination.
- If rapid tear break-up time, add a lipid-containing eye drop or spray.
- If persistent surface inflammation, add an anti-inflammatory eye drop.
- Consider oral pilocarpine to stimulate secretions.

## 3. Management of Dry Mouth

There are numerous salivary substitutes available, but no good evidence that any are superior to another [39]. In general, we recommend that patients use a sugar-free, pH-neutral oral spray or gel for comfort. Many prefer to simply drink plain water or swill the mouth with a homemade mouthwash (1 tsp bicarbonate + ½ tsp salt in a pint of boiled water cooled). Chewing gum can be used to stimulate saliva production in those with some residual glandular function, but it is not tolerated by all, as it can increase gastric acid secretion.

An array of non-drug therapies has been trialled for oral sicca [40] including acupuncture, electrostimulation, and oil pulling [41], and powered versus manual toothbrushing, but none have been shown to have a significant benefit for the symptoms of a dry mouth in trials. We do recommend that patients switch to a powered toothbrush as they are more efficient at cleaning and simple to use.

### 3.1. Stimulatory Treatments for Oral Sicca

Pilocarpine can provoke a significant improvement in symptoms of oral dryness (*p* < 0.0001) and salivary flow rates (*p* < 0.0001), but as mentioned above, adverse effects are common, with sweating (43%), flushing (10%), and urinary frequency (10%) being the most troublesome [33,34,35].

Cevimeline improves oral dryness (*p* = 0.0004) and salivary flow rates (*p* = 0.007) [36,42,43,44] but significant nausea and sweating may limit its use. It is available in the US (licenced only for adults) but not in Europe or the UK.

A head-to-head study comparing pilocarpine and cevimeline suggested similar efficacy but better tolerability for cevimeline, with less sweating (11% vs. 25%) and better perseverance as a result [45].

### 3.2. Immunotherapy for Salivary Gland Disease

Conventional anti-rheumatic drugs have not been shown to improve the sicca symptoms in SjD. No improvement in sicca symptoms or signs were seen in the few published studies of methotrexate, azathioprine, and hydroxychloroquine in Sjogren’s disease (SjD) [46]. Two small studies of leflunomide showed a reduction in systemic disease activity and an improvement in oral dryness [47,48]. A systematic review of 17 studies evaluating the immunobiologic therapies for salivary gland disease in SjD, focusing on xerostomia and salivary dysfunction, found that rituximab, abatacept, and belimumab may improve objective salivary gland function, but their effects on subjective xerostomia are inconsistent. Rituximab showed the most evidence for enhancing salivary flow, while abatacept and belimumab demonstrated potential benefits for both salivary flow and dryness perception in some studies [49]. A number of phase II and phase III trials of novel biologic agents are currently underway, with an expanding understanding of the pathophysiology of SjD opening up new potential treatment pathways [50]. One of these agents, Ianalumab, which depletes both circulating B-cells and BAFF (B cell activating factor), has recently reported to have sustained efficacy over a 52-week trial period with improvements in patient-reported dryness and stimulated salivary flow rates with a good safety profile [51] offering promise for future effective treatments. The role of immunotherapies is covered in detail elsewhere in this issue.

### 3.3. Preventing Dental Caries and Gum Disease

The permanent dentition erupts from the age of six years, and consequently, most of the published evidence on caries prevention relates to children and adolescents with no specific studies in a SjD population. Fluoride has an established role in caries prevention [52]. Studies on water fluoridation from pre 1975 indicated that it was effective at reducing caries in permanent dentition, but a contemporary review found smaller effect sizes, possibly reflecting increased use of fluoride toothpastes [53]. Fluoride-containing toothpaste outperforms non-fluoride toothpaste in preventing caries with a dose-response effect [54], and fluoride-containing mouth rinses reduce caries in permanent dentition [55]. Fluoride varnishes have been confirmed to have a substantial caries-inhibiting effect in children and adolescents [56]. Interdental cleaning is essential to prevent gingivitis and plaque build-up and assist in caries prevention; with some evidence that interdental brushes may be more effective than flossing [57].

Xylitol is a natural sweetener derived from the bark of birch trees or, more usually, commercially from corn cobs. It has a low glycaemic index, is safe for diabetics, and can be used as a sugar substitute. It has the dual benefit of improving moisture and reducing oral streptococcus mutans carriage, and its use in caries prevention in children is supported by a 2015 Cochrane review [58] and by a more recent meta-analysis [59]. In contrast, a Cochrane review of chlorhexidine found little evidence of benefit compared to a placebo [60].

Frequent sugar intake accelerates dental decay [61] and we therefore recommend that patients avoid snacking between meals, avoid sugared or carbonated drinks altogether, finish meals with tooth-friendly foods such as cheese and nuts, and use plain water and xylitol-containing products for symptomatic relief. We encourage patients to practice good dental hygiene and attend regular dental appointments where possible.

### 3.4. Summary Management Recommendations for Oral Sicca

- Excellent oral hygiene.
- Limit sugar intake and avoid eating between meals.
- Plain water between meals and overnight.
- Regular dental/hygienist visits with proactive intervention.
- Twice daily brushing with high fluoride toothpaste.
- Daily interdental cleaning.
- pH-neutral oral mousse or gel at night.
- Xylitol-containing sweets or gum.
- Consider pilocarpine to stimulate secretions.

## 4. Management of Systemic Dryness

Dryness of the bronchial mucosa contributes to the common symptom of dry cough, and an unexplained cough may be a presenting feature of SjD [62]. Reduction in voice quality is linked to the dryness of the vocal cords. Both may be helped by pilocarpine and humidification of room air. There is anecdotal evidence supporting the use of carbocisteine, a mucolytic, in patients with a dry or sticky cough and hoarseness.

Vaginal dryness is a common problem in SjD [63] and was reported by 53% individuals with SjD compared to 28% of controls *p* = 0.005 [64]. It has a significant impact on the quality of life with patients with SjD presenting with significantly higher rates of vaginal and vulvar dryness and dyspareunia compared to healthy controls [65]. Despite this, there are no published studies on management specific to individuals with SjD, and our advice is thus extrapolated from general literature. Topical oestrogens are safe and effective for vaginal atrophy in post-menopausal women [66] with equal efficacy between creams, pessaries, and intravaginal tablets. There is published evidence supporting the use of hyaluronic acid-based vaginal lubricants for vaginal dryness and dyspareunia [67]. In patients with SjD, we recommend that these are combined with non-hormonal pH-balanced (acidic) vaginal moisturisers, which are safe and effective for symptomatic relief of vaginal dryness [68,69] and are routinely recommended in guidelines [70,71].

Skin manifestations are common in SjD [72] with dermatitis in 34%, sub-acute cutaneous lupus erythematosus (SCLE) rash in 21.6%, leucocytoclastic vasculitis in 16.8%, and non-scarring alopecia in 10.3% of a Ro+ve cohort. Photosensitivity was reported by 27.6% and Raynaud’s by 26.5%. Other skin manifestations include annular erythema and livedo reticularis. Treating the underlying disease activity with hydroxychloroquine can be helpful in managing many of these manifestations, including SCLE [73] and leucocytoclastic vasculitis, and may help alopecia [74]. Dermatologists recommend a range of interventions for immune-mediated alopecia, including potent topical steroids, intralesional steroids, other pharmacotherapies, and immunosuppression, but with variable outcomes [75]. Good sun protection is important, especially for the Ro+ve patients. Raynaud’s may respond to lifestyle changes and vasodilators.

Patients frequently complain of dry skin and hair. Topical moisturisers are helpful, widely used, and freely available over the counter.

## 5. Lifestyle Advice

In addition to medical management, lifestyle advice is important. Low environmental humidity accelerates tear evaporation and exacerbates dry mouth and cough. Low humidity exacerbates dry eye symptoms [76]. Healthy workers experience dry eyes at humidity levels below 20%. Wearing glasses has been shown to reduce surface evaporation by up to 30%, and avoiding excess screen time also helps. There is some evidence supporting Omega-3 supplementation, particularly for the ocular symptoms and signs [77]. Studies of supervised exercise in SjD have shown improvements in fatigue and pain [78,79] and higher levels of physical activity were significantly associated with better health-related QOL in a cohort of 245 patients with SjD [80]. SjD is a chronic disease with no universally effective therapies, and patients may benefit from the support provided by patient organisations and programmes designed to support those with chronic disease.

## 6. Conclusions

The sicca symptoms associated with SjD are often poorly managed, resulting in a significant impact on quality of life. We advise that sicca is proactively managed in each individual patient by educating patients on self-help measures and recommending appropriate replacement and stimulatory treatments.

## Figures and Tables

**Table 1:** Suggested sequence of eye drops for progressive sicca symptoms.

Step 1	Step 2	Step 3 (If Ongoing Discomfort)	If Rapid TBUT	If Poor Tear Retention	If Persistent Surface Inflammation
0.1% Sodium hyaluronate drop (preservative-free) qds	Increase %age of Sodium hyaluronate	Combined drop, e.g., Sodium hyaluronate plus trehalose	Consider a PVA * drop	Consider a carmellose-containing drop	Consider a topical anti-inflammatory drop
Ointment at night	Ointment at night	Ointment at night	Ointment at night	Ointment at night	Ointment at night

* PVA = Polyvinylalcohol.

## Data Availability

No new data were created or analysed in this study. Data is not applicable to this article.
